# Supplementary material for: PIN1-SUMO2/3 motif suppresses excessive RNF168 chromatin accumulation and ubiquitin signaling to promote IR resistance
Source: Nat Commun. 2025 Apr 14;16:3399. doi: 10.1038/s41467-025-56974-9 (PMC11997057; doi:10.1038/s41467-025-56974-9)
Supplement: Supplementary file 1 — Supplementary information [file 41467_2025_56974_MOESM1_ESM.pdf]

Tables

Supplementary Table 1

Details of primer sequences

Name	Cloning and mutagenesis primer
myc-RNF168-Fwd	AAAGGATCCATGGAACAAAAACTCATCTCAGAAGAGGATCTGGCTCTACCCAAAGACGCCATCC
myc-RNF168-Rev	AAACTCGAGTTACTTTGTGCATCTCTGAAACATCTGAAAAACAC
myc-RNF168- Δ190-235-Fwd	AGCATTGATATTAACAATTTTGGGTCAGCCTCACAC
myc-RNF168- Δ190-235-Rev	GTGTGAGGCTGACCCAAAATTGTTAATATCAATGCT
myc-RNF168-T208A-Fwd	GAAAATCTGATCCAGTTGCACCCAAGTCTGAAAAG
myc-RNF168-T208A-Rev	CTTTTCAGACTTGGGTGCAACTGGATCAGATTTTC
myc-RNF168-P209A-Fwd	CTGATCCAGTTACAGCCAAGTCTGAAAAGAAAAG
myc-RNF168-P209A-Rev	CTTTTCTTTTCAGACTTGGCTGTAACTGGATCAG
myc-RNF168-S197A-Fwd	GGAAGTATCTCGGCTGCTCCCTTGAATTCC
myc-RNF168-S197A-Rev	GGAATTCAAGGGAGCAGCCGAGATACTTCC
myc-RNF168-T230A-Fwd	ATTCAGAAGTATTTGGCACCGAAATCTCAG
myc-RNF168-T230A-Rev	CTGAGATTTCGGTGCCAAATACTTCTGAAT
myc-RNF168-K210R-Fwd	GATCCAGTTACACCCAGGTCTGAAAAGAAAAG
myc-RNF168-K210R-Rev	CTTTTCTTTTCAGACCTGGGTGTAACTGGATC
myc-RNF168-T208A/P209A-Fwd	GAAAATCTGATCCAGTTGCAGCCAAGTCTGAAAAG
myc-RNF168-T208A/P209A-Rev	CTTTTCAGACTTGGCTGCAACTGGATCAGATTTTC
myc-RNF168-P209A/K210R-Fwd	CTGATCCAGTTACAGCCAGGTCTGAAAAGAAAAG
myc-RNF168-P209A/K210R-Rev	CTTTTCTTTTCAGACCTGGCTGTAACTGGATCAG

Supplementary Table 2

Details of siRNA sequences

siRNA	Sequence
NTC	Sense: CUUACGCUGAGUACUUCGA[dT][dT]<br>Antisense: [Phos]UCGAAGUACUCAGCGUAA G[dT][dT]
RNF168 UTR-A	Sense: GCUGCUCUCUAGGCACACUCA[dT][dT]<br>Antisense: [Phos]UGAGUGUGCCUAGAGAGCAGC[dT][dT]
RNF168 UTR-B	Sense: GUUUCCCUUCUUAAUAACAGA[dT][dT]<br>Antisense: [Phos]UCUGUUAUUAAGAAGGGAAAC[dT][dT]
PIN-A	Sense: GCUCAGGCCGCCGAGUGUACUA [dT][dT]<br>Antisense: [Phos]UAGUACACUCGGCGGCCUGAGC [dT][dT]
PIN1-B	Sense: GAAGACGCCUCGUUUGCGC [dT][dT]<br>Antisense: [Phos] GCGCAAACGAGGCGUCUUC [dT][dT]
SUMO1 UTR	Sense: CCUUCAUAUUACCCUCUCCUU[dT][dT]<br>Antisense: [Phos]AAGGAGAGGGUAAUAUGAAGG[dT][dT]
SUMO1 Ex2	Sense: CUCAAAGUCAUUGGACAGGAU[dT][dT]<br>Antisense: [Phos]AUCCUGUCCAAUGACUUUGAG[dT][dT]
SUMO2 UTR-A	Sense: GUACGUAGCUGUUACAUGU[dT][dT]<br>Antisense: [Phos]ACAUGUAACAGCUACGUAC[dT][dT]
SUMO2 UTR-B	Sense: GCGUCUUGUUGUUUAAAUA[dT][dT]<br>Antisense: [Phos]UAUUUAAACAACAAGACGC[dT][dT]

SUMO3 UTR	Sense: GGGAUGAAUCUGUAACUUA[dT][dT]<br>Antisense: [Phos]UAAGUUACAGAUUCAUCCC[dT][dT]
SUMO3 Ex2	Sense: GCAAGCUGAUGAAGGCCUA[dT][dT]<br>Antisense: [Phos]UAGGCCUUCAUCAGCUUGC[dT][dT]
53BP1 Ex1	Sense: GAUACUCCUUGCCUGAUAAUU[dT][dT]<br>Antisense: [Phos]AAUUAUCAGGCAAGGAGUAUC[dT][dT]
53BP1 UTR-A	Sense: GAUACUUGGUCUUACUGGUUU [dT][dT]<br>Antisense: [Phos]AAACCAGUAAGACCAAGUAUC[dT][dT]
RNF8 Ex3	Sense: UGGAGCAACUAGAGAAGACUU[dT][dT]<br>Antisense: [Phos]AAGUCUUCUCUAGUUGCUCCA[dT][dT]
RNF8 Ex5	Sense: CAAAGAAUUAGAGCAGACCAA[dT][dT]<br>Antisense: [Phos]UUGGUCUGCUCUAAUUCUUUG[dT][dT]
BRCA1 UTR-1	Sense: GCUCCUCUCACUCUUCAGU[dTdT]<br>Antisense: [Phos]ACUGAAGAGUGAGAGGAGC[dT][dT]
BRCA1 UTR-2	Sense: AAG CUCCUCUCACUCUUCAGU[dT][dT]<br>Antisense: [Phos]ACUGAAGAGUGAGAGGAGCUU[dT][dT]
CDK1	siRNA SMARTPool, Horizon Discovery, Cat. # L-003224-00-0010
CDK2	siRNA SMARTPool, Horizon Discovery, Cat. # L-003236-00-0010
JNK1	siRNA SMARTPool, Horizon Discovery, Cat. # L-003514-00-0010
JNK2	siRNA SMARTPool, Horizon Discovery, Cat. # L-003505-00-0010
GSK3A	siRNA SMARTPool, Horizon Discovery, Cat. # L-003009-00-0010
GSK3B	siRNA SMARTPool, Horizon Discovery, Cat. # L-003010-00-0010
TRIP12	siRNA SMARTPool, Horizon Discovery, Cat. # L-007182-00-0010
UBR5	siRNA SMARTPool, Horizon Discovery, Cat. # L-007189-00-0010
RNF4	siRNA SMARTPool, Horizon Discovery, Cat. # L-006557-00-0010

Supplementary Table 3

Details of antibody and concentration used

Antibody	Animal	Source	Cat. number	Technique	Concentration
RNF168	Rabbit	Millipore	ABE367	WB<br>IF	1:1000<br>1:500
pT208-RNF168	Mouse	Custom generated	N/A	WB	1:1000
PIN1	Mouse	R&D Systems	MAB2294	WB	1:2000
Histone H3	Rabbit	Abcam	ab1791	WB	1:2000
γH2AX	Rabbit	Abcam	ab2893	WB<br>IF	1:1000<br>1:2000
γH2AX	Mouse	Abcam	ab22551	IF	1:2000
H2AX	Rabbit	Abcam	ab11175	WB	1:1000
SUMO1	Mouse	Millipore	MABS2071	WB	1:500
SUMO2/3	Mouse	Abcam	ab81371	WB	1:1000
RNF8	Rabbit	Abcam	ab128872	WB	1:1000
Tubulin	Mouse	Santa Cruz	sc-5286	WB	1:1000
Vinculin	Rabbit	Abcam	ab129002	WB	1:2000
Vinculin	Mouse	Proteintech	66305-1-Ig	WB	1:5000
myc	Mouse	CST	2276	WB	1:1000
myc	Mouse	Merck	M5546	IF	1:500
mouse-HRP	Rabbit	DAKO	P0161	WB	1:5000
Rabbit-HRP	Swine	DAKO	P0217	WB	1:5000
53BP1	Rabbit	Abcam	ab36823	IF	1:3000
53BP1	Rabbit	Novus	NB100-904	WB	1:2000
GFP	Rabbit	Abcam		WB	1:1000
BRCA1	Mouse	Santa Cruz	Sc-6954	IF	1:500

Rad51	Rabbit	Calbiochem	PC130	IF	1:500
CENPF	Rabbit	Merck	HPA052382	IF	1:1000
CENPF	Mouse	BD	610768	IF	1:1000
CDK1	Rabbit	Proteintech	19532-1-AP	WB	1:1000
CDK2	Rabbit	Proteintech	10122-1-AP	WB	1:1000
JNK1	Mouse	CST	3708S	WB	1:1000
JNK2	Rabbit	CST	9258S	WB	1:1000
GSK-3α/β	Rabbit	CST	5676S	WB	1:1000
UBR5	Mouse	Proteintech	66937-1-IG	WB	1:5000
TRIP12	Rabbit	Bethyl Laboratories	A301-814A	WB	1:1000
H2AK15ub	Mouse	Merck	MABE1119	WB	1:1000
Cyclin A	Rabbit	Santa Cruz	Sc-751	WB	1:500
p-Histone H3 Antibody (Ser 10)	Rabbit	Santa Cruz	Sc-8656	WB	1:1000
HA	Mouse	Merck	H9658	WB	1:1000
p97/VCP	Mouse	Abcam	Ab11433	WB	1:1000
mouse-Alexa-488	Donkey	Life technologies	A21202	IF	1:2000
mouse Alexa-555	Donkey	Life technologies	A31570	IF	1:2000
Rabbit Alexa-488	Donkey	Life technologies	A21206	IF	1:2000
Rabbit Alexa-555	Donkey	Life technologies	A31572	IF	1:2000

Supplementary Table 4

Inhibitors	Source	Cat. number	Target	Concentration
Juglone	Merck	H47003	PIN1	10µM
PiB	Merck	B7688	PIN1	25µM
ATRA	Merck	R2625	PIN1	25µM
R03306	Selleckchem	S7747	CDK1/2	10µM
Roscovitine	Merck	R7772	CDK1/2	25µM
SP600125	Merck	S5567	JNK1/2	25µM
GSK-3 inhibitor IX	Merck	361550	GSK-3α/β inhibitor	5µM
INDY	Merck	405273	DYRK family	20µM
U0126	Merck	662005	MEK1/2	10µM
MG132	Merck	SML0430	Proteasome	10-20µM
CB-5083	Selleckchem	S8101	p97/VCP	1µM

Supplementary Table 5

List of synthetic peptides used for the PIN1 binding assay.

Abbreviations: Ac – Acetylation and pT – phosphorylated threonine.

Name of Peptide	Amino acid sequence	MW (Da)
T208	Ac-SDPVTPK-Amide	783
pT208	Ac-SDPV(pT)PK-Amide	864

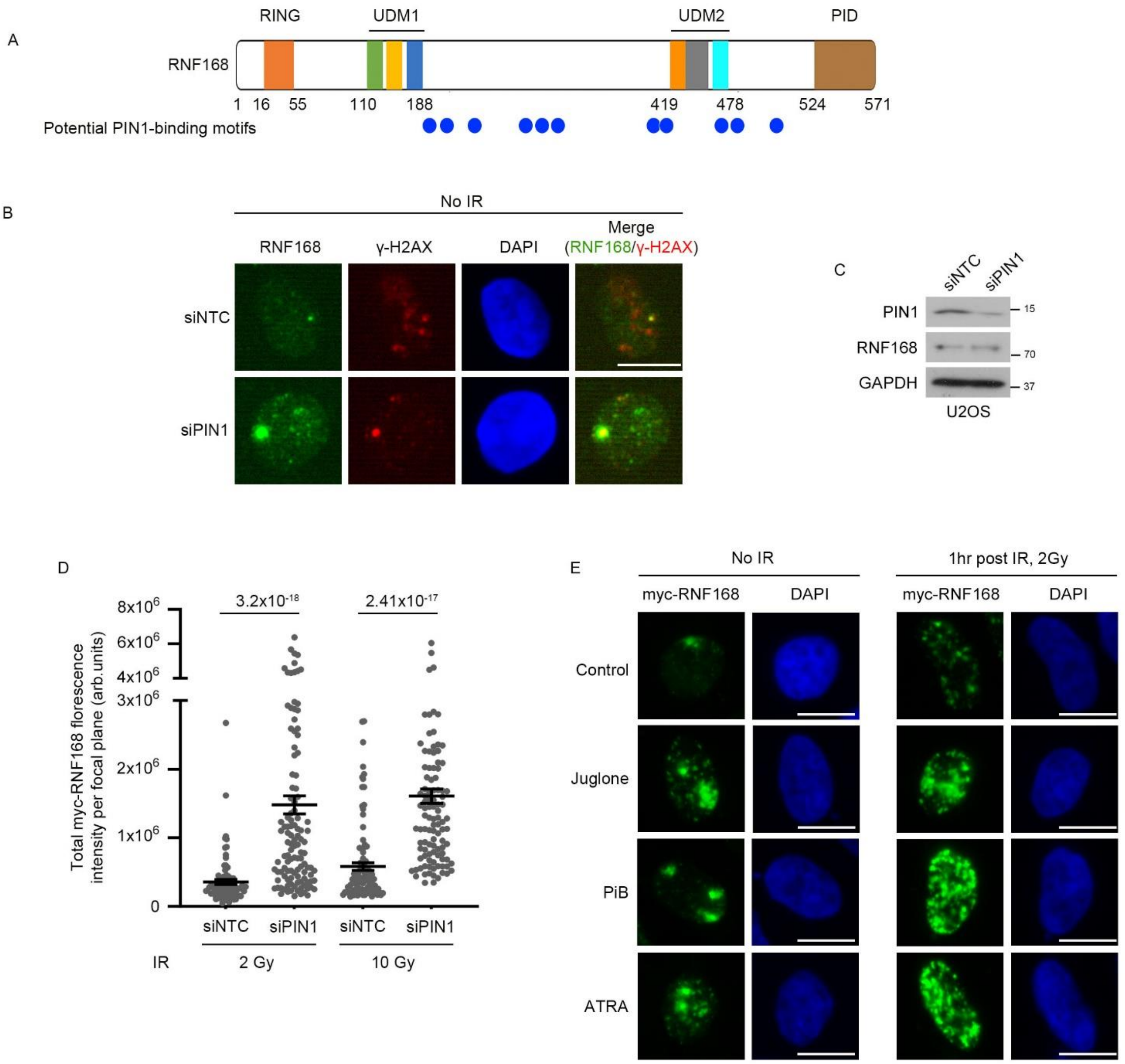

Supplementary Figure 1.

A. Diagram depicting domain architecture of RNF168. The location of potential PIN1 binding motifs in RNF168 are highlighted with blue circles.

B. Representative images U2OS cells treated with siNTC and siPIN1. Cells were stained for RNF168 and γH2AX. Scale bars 10 μm.

C. Western blot for PIN1 depletion following treatment with siNTC and siPIN1 (performed once). Source data are provided as a Source Data file.

D. Quantification of myc-RNF168 foci intensity after radiation. U2OS cells expressing myc-RNF168 were treated with siNTC or siPIN1 and stained for myc, 1 hr post IR (2 Gy and 10 Gy). myc intensity on y-axis. Data is mean ± s.e.m, n= 90 cells for siNTC (2 Gy), 120 for siPIN1 (2 Gy), 101 for siNTC (10 Gy) and 114 for siPIN1 (10 Gy). Source data are provided as a Source Data file.

E. Representative images of U2OS cells expressing myc-RNF168 and treated with Juglone (10µM, 4 hrs), PiB (25µM, 24 hrs), ATRA (25µM, 24 hrs). Cells were either left untreated or treated with 2 Gy IR. 1 hr post IR, the cells were stained for myc. Scale bars 10 µm.

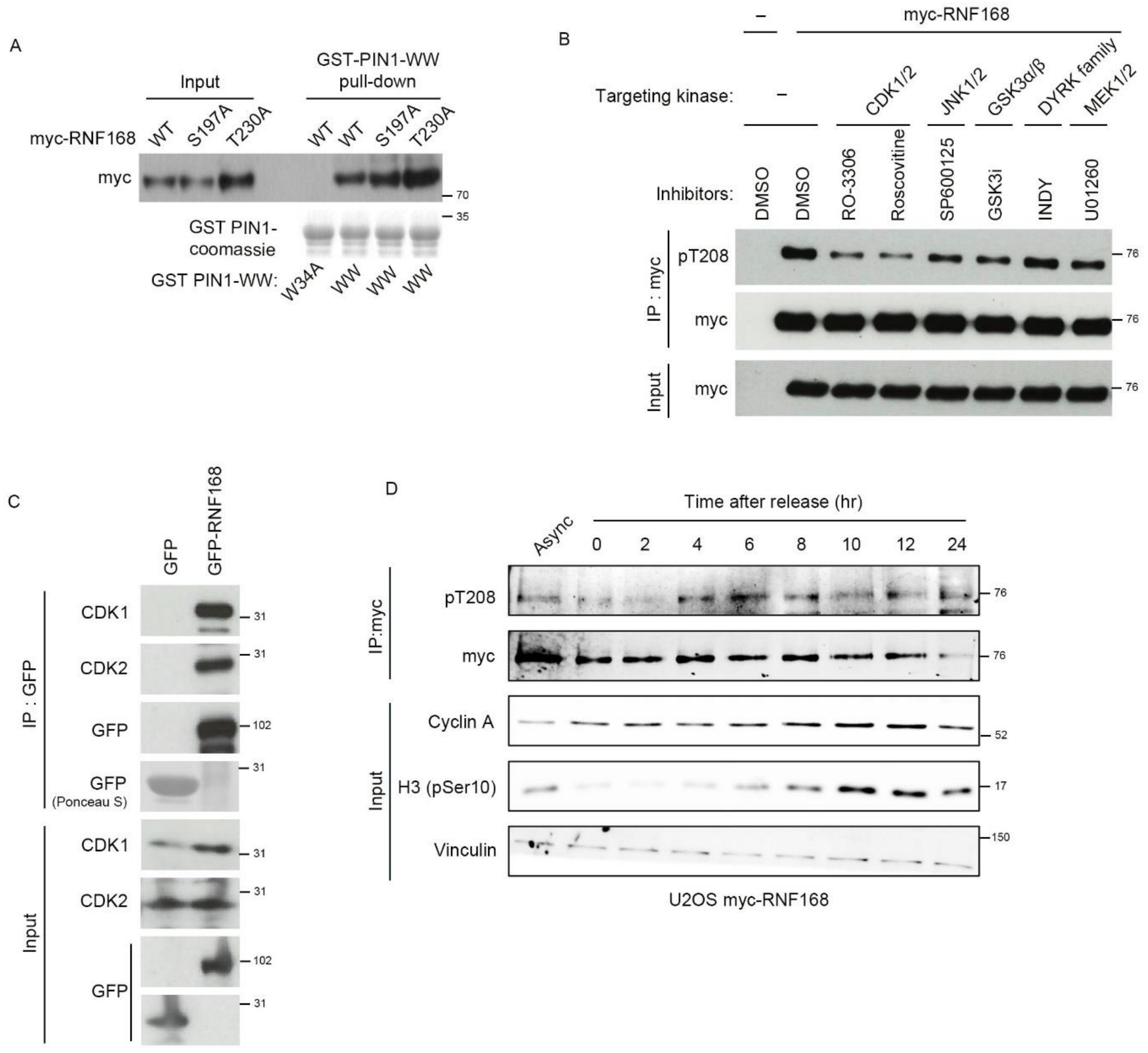

Supplementary Figure 2.

A. U2OS cells expressing myc-RNF168 WT or S197A or T230A mutant were subjected to pull down by GST-fused-WW or W34A domain of PIN1 (performed once). Source data are provided as a Source Data file.

B. HEK293 cells expressing myc-RNF168 were treated with RO-3306 (10 µM), roscovitine (25 µM), SP 600125 (25 µM), GSK-3 Inhibitor IX (5 µM), INDY (20 µM), U0126 (10 µM) for 4 hrs and probed with phospho-Thr^{208}-RNF168 and myc Ab (performed twice). Source data are provided as a Source Data file.

C. GFP-Trap precipitation of endogenous CDK1 and CDK2 with GFP and GFP-RNF168 from HEK293 cells (performed once). Source data are provided as a Source Data file.

D. Western blot for phospho-Thr^{208}-RNF168, myc, cyclin A, phospho-Ser^{10}-H3 and vinculin from myc-RNF168-U2OS cells after release at indicated time points following double thymidine block (performed once). Source data are provided as a Source Data file.

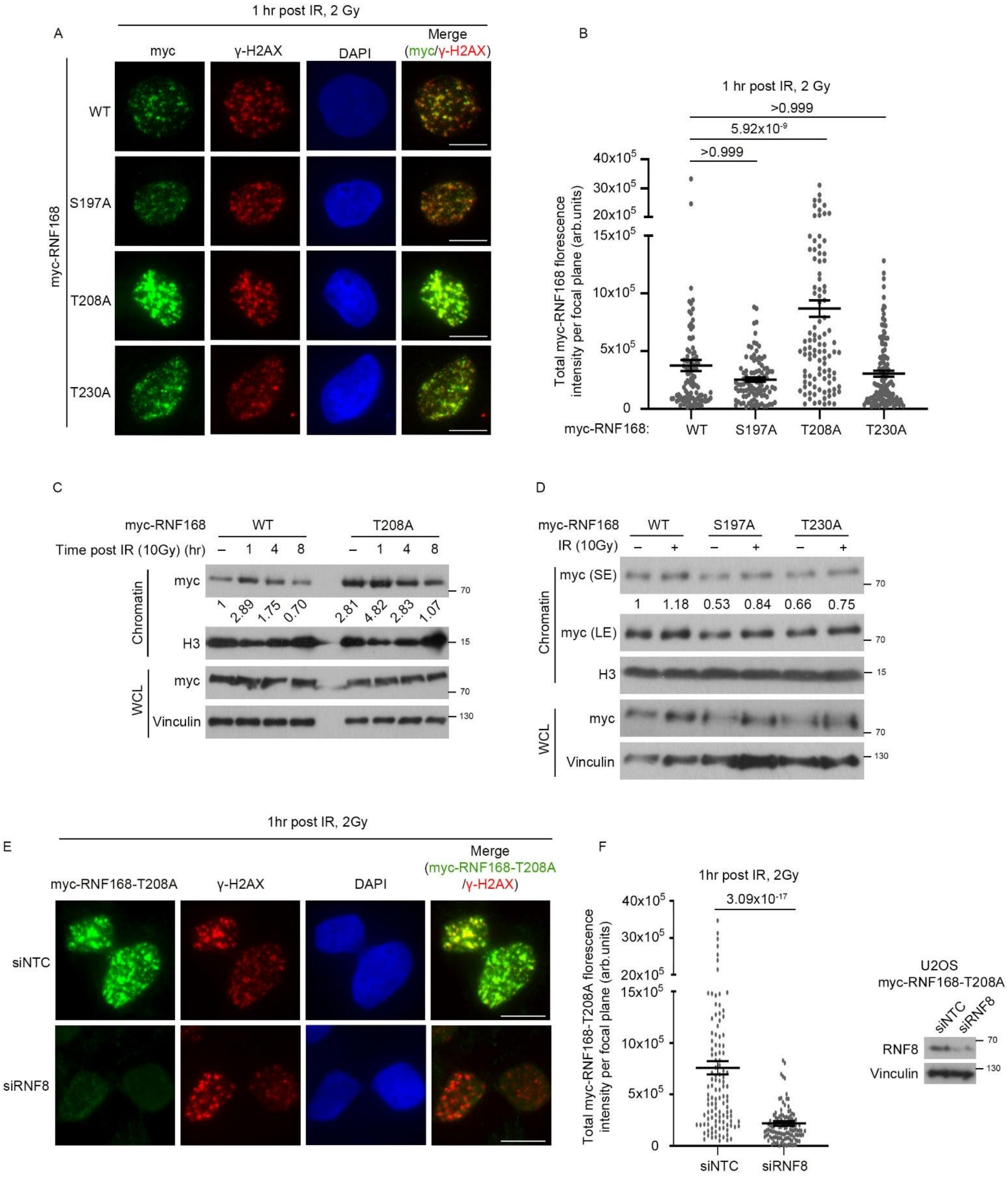
A
1 hr post IR, 2 Gy
myc
γ-H2AX
DAPI
Merge (myc/γ-H2AX)
myc-RNF168
WT
S197A
T208A
T230A
B
1 hr post IR, 2 Gy
>0.999
5.92x10-9
>0.999
Total myc-RNF168 florescence intensity per focal plane (arb.units)
40x10^5
30x10^5
20x10^5
15x10^5
10x10^5
5x10^5
0
myc-RNF168:
WT
S197A
T208A
T230A
C
myc-RNF168
WT
T208A
Time post IR (10Gy) (hr)
– 1 4 8
– 1 4 8
Chromatin
myc
1 2.89 1.75 0.70 2.81 4.82 2.83 1.07
H3
WCL
myc
Vinculin
70
15
70
130
D
myc-RNF168
WT
S197A
T230A
IR (10Gy)
– + – + – +
Chromatin
myc (SE)
1 1.18 0.53 0.84 0.66 0.75
myc (LE)
H3
WCL
myc
Vinculin
70
70
15
70
130
E
1hr post IR, 2Gy
myc-RNF168-T208A
γ-H2AX
DAPI
Merge (myc-RNF168-T208A /γ-H2AX)
siNTC
siRNF8
F
1hr post IR, 2Gy
3.09x10-17
Total myc-RNF168-T208A florescence intensity per focal plane (arb.units)
40x10^5
30x10^5
20x10^5
15x10^5
10x10^5
5x10^5
0
siNTC
siRNF8
U2OS
myc-RNF168-T208A
siNTC
siRNF8
RNF8
Vinculin
70
130

Supplementary Figure 3.

A. Representative images of RNF168 mutant foci. U2OS cells stably expressing myc-RNF168-WT, S197A, T208A and T230A were fixed 1 hr after IR exposure (2 Gy) and stained for myc and γ-H2AX. Scale bars 10 µm.

B. Quantification of foci intensity from A. Data is mean ± s.e.m, n= 102 for WT, 98 for S197A, 111 for T208A, 120 for T230A. Source data are provided as a Source Data file.

C. Western blot of chromatin fraction and WCL for myc, histone H3 and vinculin. U2OS cells complemented with RNF168-WT or T208A variant were treated with 10 Gy IR. Cells were collected at various time points post-IR for fractionation (performed twice). Source data are provided as a Source Data file.

D. Western blot of chromatin fraction and WCL for myc, histone H3 and vinculin. U2OS cells complemented with RNF168-WT, S197A and T230A were treated with 10 Gy of IR. 1 hr later, untreated or IR-treated cells were collected for fractionation (performed twice). Source data are provided as a Source Data file.

E. U2OS cells complemented with myc-RNF168-T208A were treated with siNTC or siRNF8. Cells were treated with 2 Gy of IR and fixed 1 hr later. Cells were stained for myc and γ-H2AX. Scale bars 10 µm.

F. Quantification of myc-RNF168-T208A intensity from E. Data is mean ± s.e.m, n> 120 cells (Left). Western blot to show depletion of RNF8 in U2OS cells complemented with myc-RNF168-T208A (Right) (performed once). Source data are provided as a Source Data file.

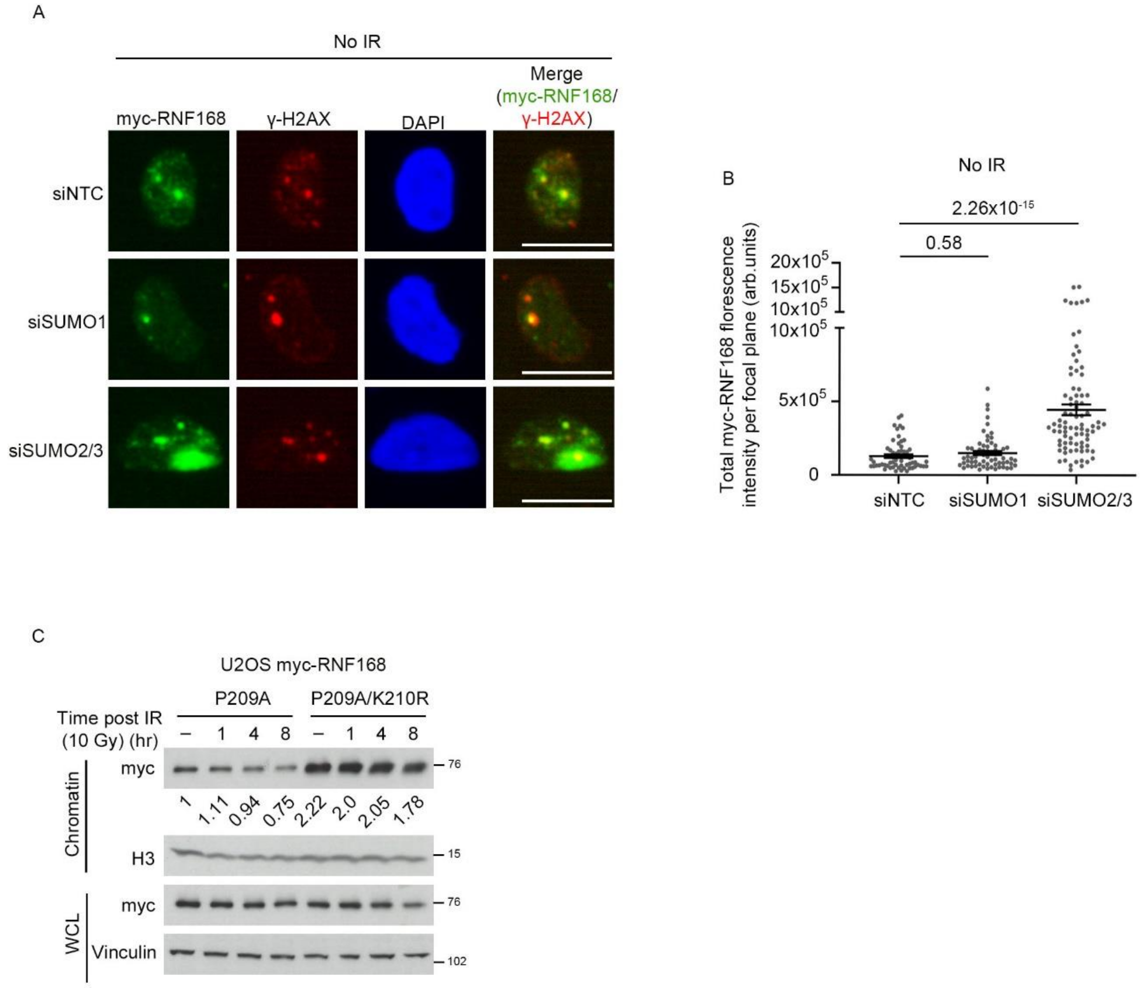

Supplementary Figure 4.

A. U2OS cells stably expressing myc-RNF168-WT were treated with indicated siRNAs and stained for myc and γ-H2AX. Scale bars 10 µm.

B. Quantification of myc-RNF168 intensity from A. Data is mean ± s.e.m, n= 61 cells for siNTC, 65 for siSUMO1 and 84 for siSUMO2/3. Source data are provided as a Source Data file.

C. Western blot of chromatin fraction and WCL for myc, histone H3 and vinculin. U2OS cells expressing RNF168-P209A or P209A/K210R mutants were treated with 10 Gy of IR and collected at indicated time points for fractionation (performed once). Source data are provided as a Source Data file.

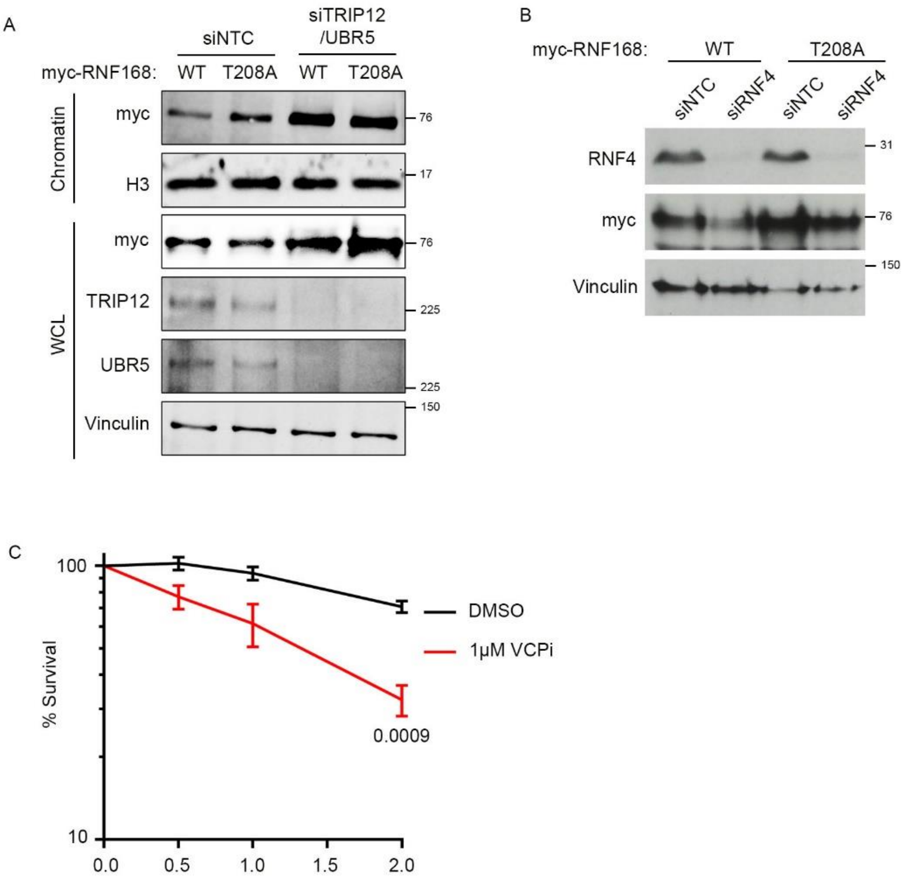

Supplementary Figure 5.

A. Western blot of chromatin fraction and WCL for myc, histone H3, TRIP12, UBR5 and vinculin. U2OS cells expressing RNF168-WT or T208A mutant were treated with siNTC or siTRIP12and siUBR5 were collected for fractionation (performed twice). Source data are provided as a Source Data file.

B. Western blot to show depletion of RNF4 in U2OS cells expressing myc-RNF168-WT or T208A mutant (performed once). Source data are provided as a Source Data file.

C. Colony survival of U2OS cells treated with indicated doses IR and p97/VCP inhibitor. 1 hr post IR, cells were treated with 1μM p97/VCP inhibitor for 24 hr. n=3. Data is mean ± s.e.m. Source data are provided as a Source Data file.

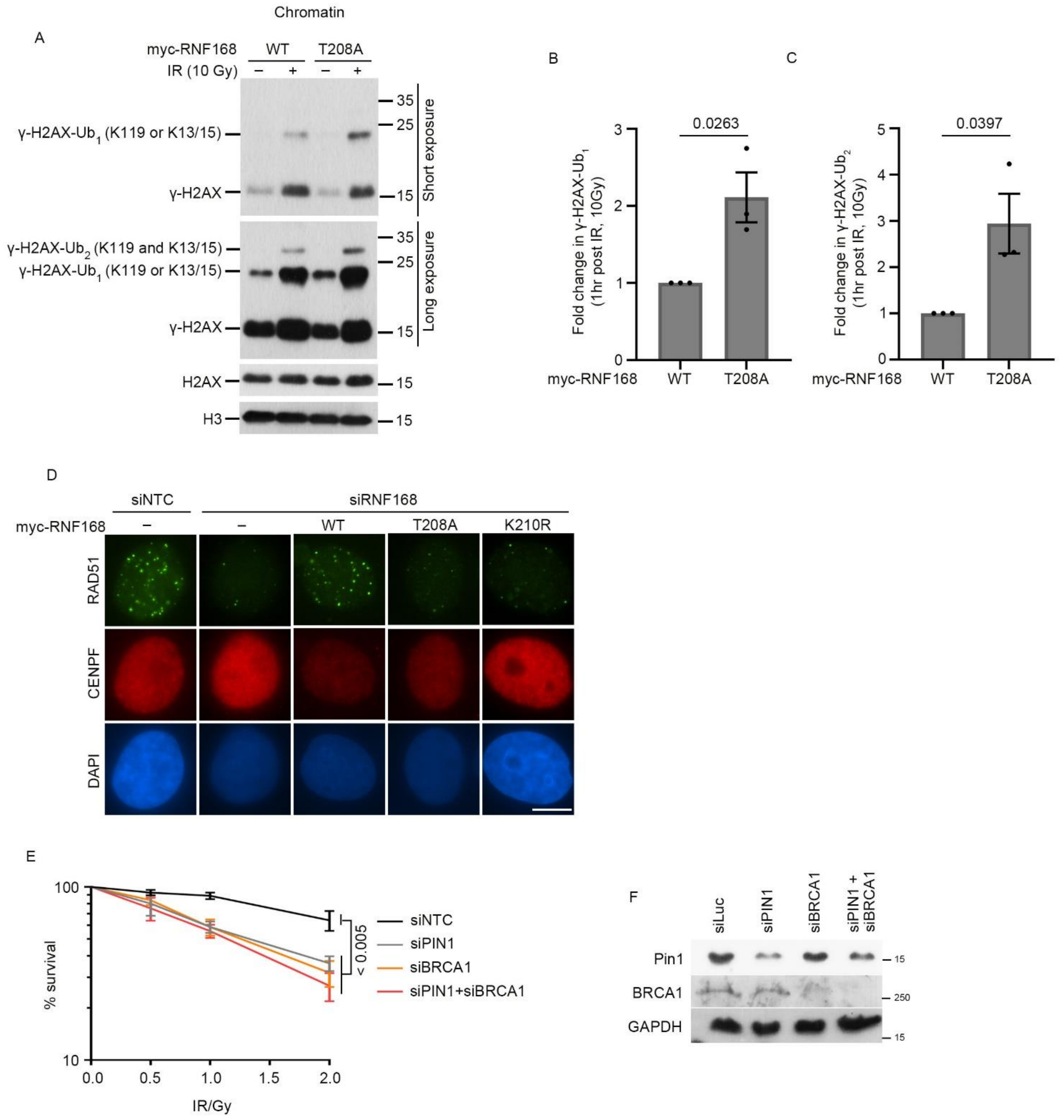

Supplementary Figure 6.

A. Western blot of chromatin fraction for γH2AX, H2AX and H3 from cells expressing siRNA-resistant myc-WT-RNF168 or T208A. Endogenous RNF168 is depleted by siRNA. Cells were either left untreated or irradiated by 10 Gy IR and fractionated 1 hr later (performed three times). Source data are provided as a Source Data file.

B. Quantification of mono-Ub- γH2AX as in A from three independent repeats. p-value <0.05. Source data are provided as a Source Data file.

C. Quantification of di-Ub-γH2AX as in A from three independent repeats. p-value <0.05. Source data are provided as a Source Data file.

D. RAD51 recruitment in cells expressing PIN1 binding (T208A) and SUMOylation (K201R) mutants of RNF168. U2OS expressing siRNA-resistant myc-RNF168-WT, T208A or K210R were depleted of endogenous RNF168 and treated with 2 Gy IR. 2 hrs post IR, cells were stained for RAD51 and CENPF. Scale bars 10 µm.

E. Colony survival of U2OS cells depleted of PIN1 or BRCA1 or both after treatment with indicated doses of IR. Mean of three independent experiments is plotted. Data is mean ± s.e.m. Source data are provided as a Source Data file.

F. Western blot to show depletion of PIN1 and BRCA1 in U2OS cells (performed once). Source data are provided as a Source Data file.
